# Immunogenicity and Protective Efficacy of a Novel Recombinant BCG Strain Overexpressing Antigens Ag85A and Ag85B

**DOI:** 10.1155/2012/563838

**Published:** 2012-04-18

**Authors:** Chun Wang, Ruiling Fu, Zhenhua Chen, Kun Tan, Lingxia Chen, Xindong Teng, Jia Lu, Chunwei Shi, Xionglin Fan

**Affiliations:** Laboratory of Biosafety, Department of Pathogen Biology, Tongji Medical College, Huazhong University of Science & Technology, No. 13, Hangkong Road, Wuhan 430030, China

## Abstract

Recombinant Bacillus Calmette-Guérin (rBCG) strain is the promising vaccine candidate for tuberculosis (TB) prevention, which aims at providing more enduring and enhanced protection than the parental BCG vaccine. In this study, three rBCG strains overexpressing immunodominant antigens Ag85B (rBCG::85B), Ag85A (rBCG::85A), or both (rBCG::AB) of *Mycobacterium tuberculosis* were constructed, respectively. rBCG strains showed higher level of overexpression of Ag85A and/or Ag85B proteins than BCG containing empty vector pMV261(rBCG::261), which had low levels of endogenous expression of both proteins as expected. rBCG::AB strain could provide the strongest short-term and long-term protection in the lung against intravenous infection with virulent *M. tuberculosis* than rBCG::261 control and other two rBCG strains overexpressing single antigen. The stronger and longer-lasting protection provided by rBCG::AB than rBCG::261 was correlated with systemic *in vitro* antigen-specific IFN-**γ** responses. Therefore, our results indicate that rBCG::AB could be a very promising TB vaccine candidate and should be further evaluated for the preclinical test.

## 1. Introduction

Tuberculosis (TB) is the second leading infectious cause of mortality worldwide. Although* Mycobacterium bovis *Bacillus Calmette-Guérin (BCG) vaccine is effective in children against miliary and meningeal TB, it provides variable protection against pulmonary TB in adults, because BCG only confers protection for 5–10 years following the neonatal vaccination and is not effective for inducing long-term cellular-mediated immunity (CMI) and protection [1]. Therefore, BCG in its current form is not a satisfactory vaccine for TB and new vaccines are urgently needed.

One important strategy in designing new TB vaccines is to develop recombinant BCG (rBCG) strains overexpressing* M. tuberculosis* antigens, which might stimulate more potent immune responses against *M. tuberculosis* infection and conferring more enduring protection than BCG vaccination. rBCG strains, which have been genetically manipulated to overexpress immunodominant antigens of *M. tuberculosis*, are promising vaccine candidates in this area, such as the rBCG strains overexpressing protective antigens Ag85B [2–5], Ag85A [6–8], Ag85C [9], ESAT-6 [10–13], or HspX [14], respectively. Moreover, these rBCG strains showed better protective efficacy than the parent BCG vaccine in experimental animal models of TB.

During the past decades, animals vaccinated with DNA or subunit vaccines based on multiple antigens showed more potent effects of preventing against *M.tuberculosis* infection than those with single antigen alone [15–19]. rBCG strain overexpressing the fusion protein of multiple antigens also would lead to increased protection against *M. tuberculosis* infection [20–25]. In the present study, we constructed an rBCG strain overexpressing antigens both Ag85A and Ag85B, and assessed its immunogenicity and protective efficacy as a vaccine against *M. tuberculosis* infection in mice.

## 2. Materials and Methods

### 2.1. Bacterial Strains and Cultures

*Escherichia coli *DH5*α* and BL21 (DE3) strains were grown in Luria-Bertani medium and used for cloning and expression, respectively. *M. bovis *BCG China (obtained from Shanghai Institute of Biological Products which is the main manufacturer of BCG vaccine in China), *M. tuberculosis* H37Rv and rBCG strains were grown in either Middlebrook 7H9 medium (Difco Laboratories, Detroit, MI) or on Middlebrook 7H11 agar (Difco Laboratories), supplemented with 10% ADC, 0.5% glycerol and 0.05% Tween 80. When required, kanamycin or ampicillin was added at a final concentration of 25 *μ*g/mL or 100 *μ*g/mL, respectively.

### 2.2. Overexpression of Recombinant Ag85A and Ag85B Proteins in *E. coli*

Recombinant Ag85A and Ag85B proteins were produced as described previously [14, 26]. Briefly, both *fbpA* and *fbpB* without their signal peptide sequences were cloned into pProExHTb (Life Technologies, Rockville, MD, USA) and constructed as recombinant plasmids pPro85A and pPro85B, respectively. Both Ag85A and Ag85B proteins with N-terminal HIS_6_ tag were expressed from *E. coli* BL21 (DE3) strains harboring recombinant plasmids and then purified on an Ni-NTA column (Life Technologies) according to the manufacturer's instructions, lyophilized and confirmed by Western blotting. Both proteins were diluted in phosphate-buffered saline (PBS) using pyrogen-free reagents and stored at −20°C. Endotoxin contamination and protein concentration were determined as described previously [14]. 

### 2.3. Construction of rBCG Strains

Two recombinant BCG strains, rBCG::85B (overexpressing antigen Ag85B) and rBCG::261 (containing empty vector pMV261), were produced as described previously [14]. The full length sequence for *fbpA* (about 1.8 kb) was amplified by PCR from *M. tuberculosis* H37Rv genomic DNA. pMAg85A was created by cloning PCR-amplified *fbpA* into the pcDNA3.1(−) vector and then subcloned into the pMV261 vector. pMAg85AB was further constructed by subcloning *fbpB* from pMAg85B into pMAg85A. Each gene was expressed from its own promoters and targeted the expressed protein for secretion through their own signal peptide sequences. Recombinant plasmids amplified in *E. coli* DH5*α* were transformed into BCG by electroporation. Kanamycin-resistant colonies were selected and grown in Middlebrook 7H9 broth. Culture supernatants from cultures that had reached an optical density at 600 nm of 1.0 were harvested after centrifugation and passage through a 0.22 *μ*m pore-size filter. Samples from supernatants were concentrated approximately 50-fold by freeze drying. Cell pellets were washed, resuspended in PBS with the equivalent values of cell density, and disrupted on ice with an Ultrasonic Processor. The bacterial cell debris from the sonicated samples was removed by centrifugation. About 40 *μ*g proteins of bacterial pellets or supernatants were separated by SDS-PAGE on a 12.5% gel and then analyzed by Western blotting. Total proteins were then transferred onto a nitrocellulose membrane. The membrane was saturated with 1% bovine serum albumin (BSA) in PBS—0.1% Tween 20 (PBST) and then incubated with anti-Ag85A chicken polyclonal antibody (diluted 1/2000; ab14073, Abcam, UK) or anti-Ag85B rabbit polyclonal antibody (diluted 1/2000; ab43019, Abcam, UK). Goat anti-chicken HRP-conjugated antibodies (Biosynthesis Biotech. Co. LTD, Beijing, China) or goat anti-rabbit HRP-conjugated antibodies (Zhongshan Goldenbridge Biotech. Co. LTD, Beijing, China) diluted 1/5000 in PBST were then used to develop the immunoblots as described [14]. The positive plasmid constructs rBCG::85A and rBCG::AB were also sequenced. The anticipated sequences of the inserts were confirmed.

### 2.4. Mice and Immunization Protocol

Animal experiments were performed in accordance with the guidelines of Chinese Council on Animal Care. The research protocol was approved by Tongji Medical School Committees on Biosaftey. Specific pathogen-free, female C57BL/6 mice at 6 weeks of age (Beijing Vital River Com., Beijing, China) were bred in separate cages in a biosafety laboratory and fed commercial mouse chow and water *ad libitum*. Twenty-two mice in each group were immunized s.c. once at the base of the tail with 1 × 10^6^ CFU of either rBCG::85A, rBCG::85B, or rBCG::AB in a final volume of 100 *μ*L of PBS. Two control groups were immunized with rBCG::261 or treated with 100 *μ*L of PBS, respectively. Six mice were sacrificed for the immunological assay after 6 or 24 weeks. Other mice were challenged with virulent *M. tuberculosis* strain 6 weeks after immunization and 5 mice were used for bacterial load in organs and pathological analysis at 10 and 24 weeks, respectively. All experiments were performed as in Figure 3 and repeated three times.

### 2.5. Antigen-Specific IgG Assay

Antigen-specific IgG antibody in the serum of each mouse was determined by enzyme-linked immunosorbent assay (ELISA). ELISA plates were coated overnight at 4°C with 100 *μ*L either Ag85B, Ag85A or antigen control (5 *μ*g/mL) in carbonate/bicarbonate buffer (pH 9.6). The plates were blocked with 200 *μ*L/well PBS containing 1% bovine serum albumin for 30 min at 37°C and washed with PBS containing 0.05% Tween 20 five times. Individual serum samples were added at serial two-fold dilutions (beginning at a 1/500 dilution) and incubated for 2 h at 37°C and washed, followed by addition of 100 *μ*L/well horseradish peroxidase-conjugated rabbit anti-mouse IgG (diluted at 1/5000). Plates were incubated for 1 h at 37°C, washed and developed with 3, 3′5, 5′-Tetramethylbenzidine (TMB) substrate. Reactions were stopped by addition of 50 *μ*L/well of 1 N H_2_SO_4_ and were read on an ELISA plate reader at 450 nm. The OD value from antigen control was subtracted as background to exclude antibody against nonspecific *E. coli* protein. Sera from mice group treated with PBS were used as the negative control (*N*). Sera with *P/N* value ≥2.1 were considered positive. Antibody titers were expressed as reciprocal end point titers and results were showed as the mean of log⁡_2_ antibody titer of each vaccinated group.

### 2.6. IFN-*γ* Enzyme-Linked Immunospot (ELISPOT) Assay

Six and 24 weeks after C57BL/6 mice were vaccinated, the mouse IFN-*γ* ELISPOT kit (U-CyTech biosciences, Netherlands) was used to determine the number of IFN-*γ* expressing cells in the single-cell spleen suspensions following the manufacturer's instructions. Lymphocytes from spleen of three mouse in each group were prepared using EZ-Sep Mouse 1 × Lymphocyte Separation Medium (Dakewe Biotech Com., Shenzhen, China) according to the manufacturer's recommendations. The cells were diluted to a concentration of 2.5 × 10^6^/mL with Lympho-Spot serum-free medium for rodent (Dakewe Biotech Com.) containing an appropriate stimulus (2 *μ*g/well of PPD, Ag85A or Ag85B). 2.5 ×10^5^ cells were added to the wells of the ELISPOT plate. IFN-*γ* spot-forming cells (SFCs) were enumerated using an ELISPOT Reader (Biosys Bioreader 4000 PRO). For each animal, the number of spots in wells with medium alone was subtracted from the number of spots in test wells. The mean number of antigen-specific IFN-*γ* spot-forming cells per million cells for each group was determined.

### 2.7. Challenge of Mice with Virulent *M. tuberculosis* H37Rv

Six weeks after immunization, ten C57BL/6 mice in each group were challenged by the injection of 10^6^ CFU of virulent *M. tuberculosis* H37Rv through a lateral tail vein. Four weeks after challenge, five mice per group were sacrificed for efficacy comparison and the remainder of infected C57BL/6 mice were kept up to 18 weeks for observation of long-term protection. The spleens and lungs were removed aseptically, homogenized, and cultured for CFU of *M. tuberculosis* on Middlebrook 7H11 agar containing 2 *μ*g/mL of 2-thiophenecarboxylic acid hydrazide (TCH, Beijing Luqiao Corp., Beijing, China) to selectively inhibit the growth of the residual BCG.

### 2.8. Histopathological Analysis

Four weeks and 18 weeks after the challenge infection, left lung lobes from different vaccine groups were fixed in 10% formalin in PBS and embedded in paraffin. Tissue sections were prepared and stained with hematoxylin and eosin (HE) stain and acid-fast stain. The results of pathological changes were recorded by a pathologist with no prior knowledge for the treatment groups under a light microscope.

### 2.9. Statistical Analysis

Student's *t*-test was used to compare the difference between groups and the difference was considered statistically significant when the *P* value was less than 0.05.

## 3. Results

### 3.1. Purification of Recombinant Ag85A and Ag85B Proteins

**Figure 1:**
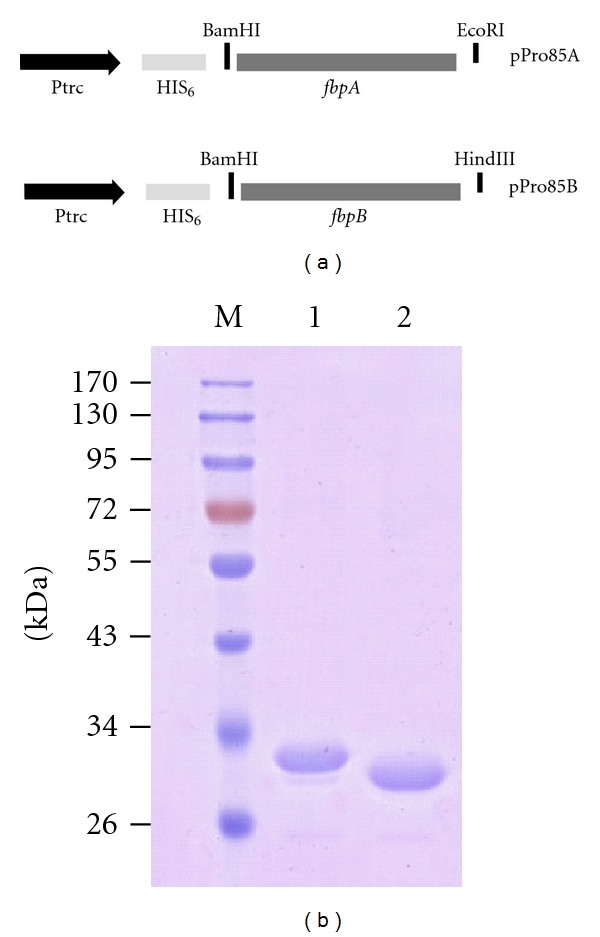
Recombinant prokaryotic expression plasmids pPro85A and pPro85B (a) and the purification of recombinant Ag85A and Ag85B proteins (b). The final products of recombinant Ag85A and Ag85B proteins were confirmed by SDS-PAGE and stained with a Coomassie blue dye. Lane M, protein molecular size marker (kDa); lane 1, the final products of Ag85A (32 kDa); lane 2, the final products of Ag85B (30 kDa).

Prokaryotic expression plasmids pPro85A and pPro85B were shown as Figure 1(a). DNA sequencing and enzyme digestion confirmed the successful constructions. *E. coli* BL21 (DE3) strains containing plasmid pPro85A or pPro85B were induced with IPTG and purified on a Ni-NTA column, respectively. The final purified products of recombinant proteins Ag85A (32 kDa) and Ag85B (30 kDa) were identified by 12% SDS-PAGE as expected (Figure 1(b)).

### 3.2. Construction and Overexpression of Ag85A and Ag85B Proteins from rBCG Strains

**Figure 2:**
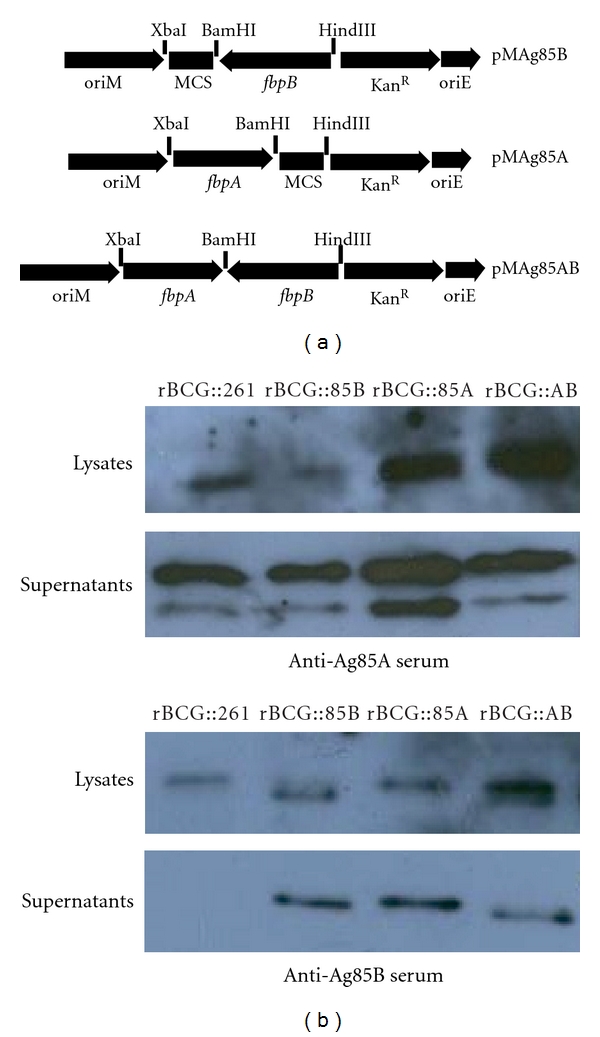
Recombinant *Mycobacterium*—*E. coli* shuttle plasmids (a) and expression of rBCG strains (b). The full-length copies of the *M. tuberculosis fbpA*(1.8 kb) or *fbpB*(1.5 kb) gene and its flanking regions including the promoter region were amplified from *M. tuberculosis* H37Rv chromosomal DNA, respectively. PCR products were digested by different enzymes and cloned into the *Mycobacterium*—*E. coli* shuttle vector pMV261. Ag85A and Ag85B proteins in the cell lysates and supernatants of rBCG::261, rBCG::85B, rBCG::85A, and rBCG::AB were detected by Western blotting analysis with anti-Ag85A or anti-Ag85B sera, respectively.

**Figure 3:**
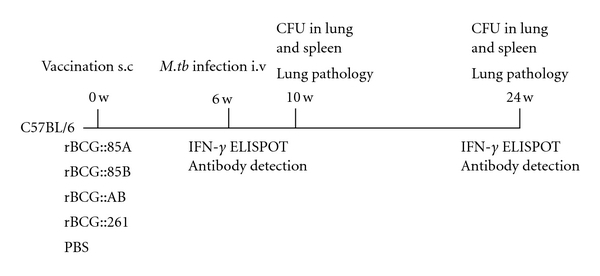
Experimental regimens used for both short-term and long-term immunological and protection studies in mice.

Recombinant *Mycobacterium*—*E. coli* shuttle plasmids pMAg85A and pMAg85AB were constructed as in Figure 2(a). Two rBCG strains, rBCG::85A and rBCG::85AB, were obtained by transformation of BCG with the recombinant plasmids pMAg85A and pMAg85AB, respectively. The overexpression of proteins in both culture supernatants and cell lysates of rBCG strains was analyzed by Western blotting (Figure 2(b)). As expected, low levels of Ag85A and Ag85B proteins were detected in empty vector control rBCG261 strain because of endogenous expression. Ag85A protein was mainly found in the culture supernatant and cell lysate of rBCG::85A and rBCG::AB strains and had a much higher concentration than those of rBCG:85B or rBCG:261 strains. rBCG::AB and rBCG::85B strains produced much more Ag85B protein in the cell lysate than rBCG::85A and rBCG:261 strains. In addition, rBCG::85A also secreted more Ag85B protein in the culture supernatant with uncertain mechanism than rBCG::85B, whatever anti-Ag85A or anti-Ag85B serum was used to detect.

### 3.3. Antibody Responses Induced by rBCG Strains

**Figure 4:**
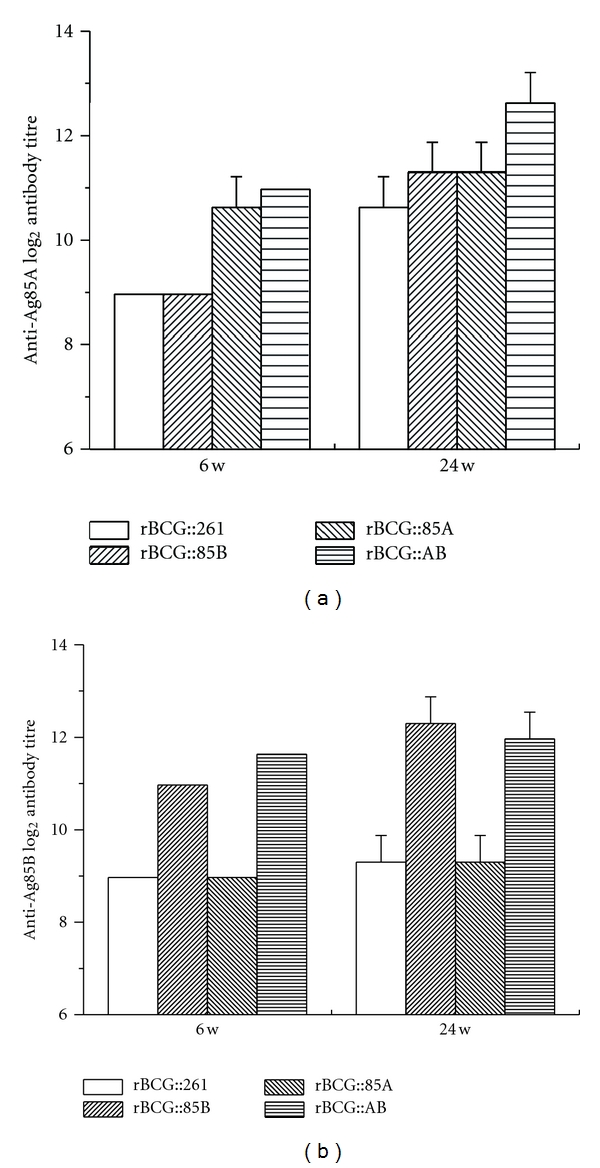
IgG antibodies against Ag85A or Ag85B in mice immunized with rBCG strains (*n* = 6). Sera were collected from each mice immunized with different rBCG strains at 6 and 24 weeks after immunization. Shown is the mean ± SEM log_2_ antibody titre per group. This experiment was repeated with similar results. At 6 w, rBCG::85A versus rBCG::261, rBCG::AB versus rBCG::261 with a *P* < 0.05 in anti-Ag85A IgG antibodies; rBCG::85B versus rBCG::261, rBCG::AB versus rBCG::261 with a *P* < 0.05 in anti-Ag85B IgG antibodies. At 24 w, rBCG::85B versus rBCG::261, rBCG::85A versus rBCG::261, and rBCG::AB versus rBCG::261 with a *P* < 0.05 in anti-Ag85A IgG antibodies; rBCG::85B versus rBCG::261, rBCG::AB versus rBCG::261 with a *P* < 0.05 in anti-Ag85B IgG antibodies. All groups induced higher levels of antibodies at 24 weeks than those did at 6 weeks (*P* < 0.05).

Antibody responses against the recombinant purified Ag85A or Ag85B protein were evaluated by ELISA in the sera of mice from different groups after 6 and 24 weeks, respectively (Figure 4). The antibody titers of the PBS control group were negative (data not shown). Mice vaccinated with different rBCG strains induced higher levels of antibody against Ag85A or Ag85B protein and IgG antibody titers increased gradually during the 6–24 weeks. rBCG::AB produced the highest antibody titres against Ag85A protein among all groups and much higher than both rBCG::85B and rBCG::261 strains at 6 and 24 weeks (*P* < 0.05). rBCG::AB also produced higher antibody titres against Ag85B than both rBCG::85A and rBCG::261 strains at 6 and 24 weeks (*P* < 0.05). 

### 3.4. IFN-*γ* ELISPOT Assay

**Figure 5:**
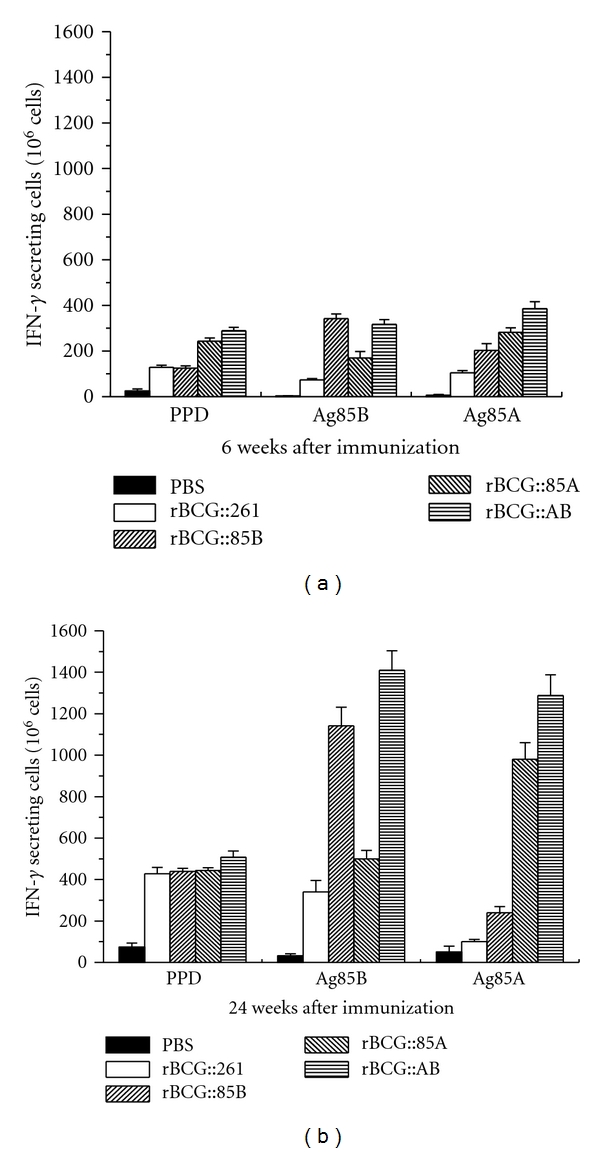
Interferon (IFN)-g secretion was measured in an ELISPOT assay with splenocytes isolated from C57BL/6 mice immunized with different rBCG strains after 6 and 24 weeks (*n* = 3). Freshly isolated spleen cells were plated in duplicate at 2.5 × 10^5^ cells per well and incubated with 2 *μ*g/well of PPD, Ag85B, Ag85A, or culture medium control for 72 h at 37°C, 5% CO_2_, respectively. Results are expressed as the mean (±SD). This experiment was repeated with similar results. There was statistic difference between all groups with different stimulations (*P* < 0.05) except rBCG::85B versus rBCG::261 with PPD stimulation at 6 and 24 weeks.

Splenocytes were isolated from mice immunized with rBCG strains following stimulation with purified Ag85B, Ag85A, or PPD, respectively (Figure 5). Splenocytes from PBS control group only produced very low number of IFN-*γ* secreted cells during the whole experimental period, whatever stimulated with PPD, Ag85A, or Ag85B proteins. Larger number of IFN-*γ* secreted cells was induced in mice vaccinated with different rBCG strains at 24 weeks than 6 week after immunization (*P* < 0.05). Upon stimulation with PPD, splenocytes from mice vaccinated with rBCG::AB produced the highest levels of IFN-*γ* than other groups at 6 and 24 weeks (*P* < 0.05). When stimulated with Ag85A, the number of IFN-*γ* secreted cells increased significantly in both rBCG::85A and rBCG::AB groups, which was much more than rBCG::261 and rBCG::85B groups at 6 and 24 weeks (*P* < 0.05). The highest number of IFN-*γ* secreting cells was from rBCG::AB group when Ag85A protein used (*P* < 0.05) (Figure 5). Both rBCG::AB and rBCG::85B groups also induced higher level of IFN-*γ* responses to Ag85B than rBCG::261 and rBCG::85A groups at 6 and 24 weeks (*P* < 0.05). 

### 3.5. Protective Efficacy of rBCG Strains

**Figure 6:**
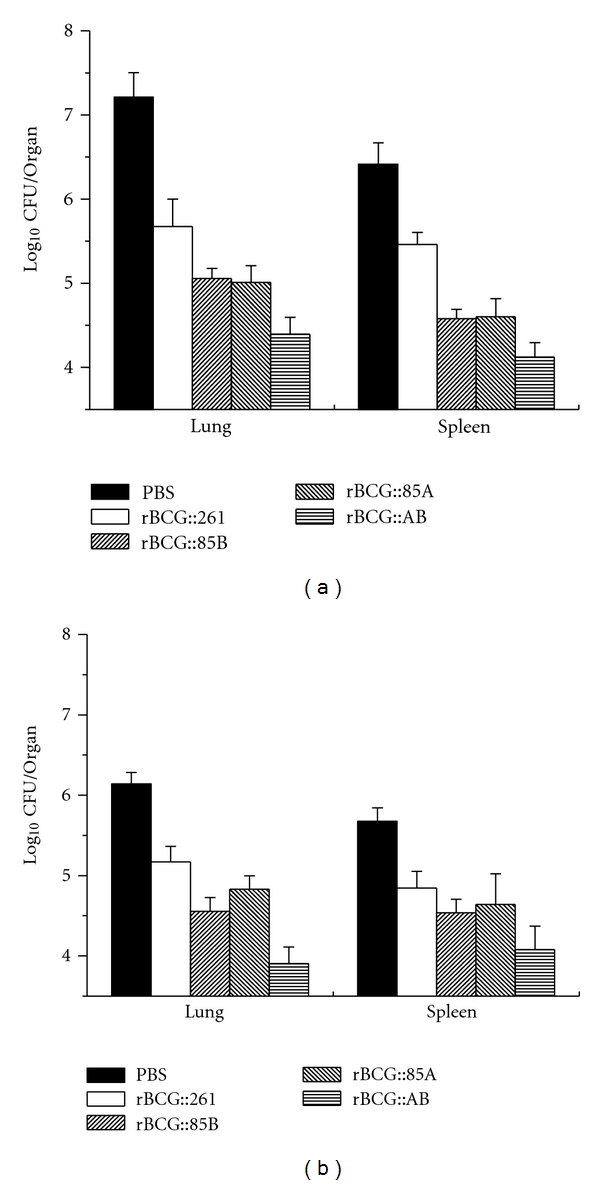
Bacterial loads per lung and spleen in C57BL/6 mice at 4 and 18 weeks after challenge. Six weeks after immunization, C57BL/6 mice (*n* = 5) were challenged i.v. with 10^6^ CFU virulent *M. tuberculosis* H37Rv strain. Four and eighteen weeks after challenge, spleens and lungs were harvested and numbers of CFU per organ were enumerated. Results are shown as the mean (±SEM) log10 CFU/organ. This experiment was repeated with similar results. At 4 w after challenge, PBS versus rBCG::261, rBCG::AB versus rBCG::261, and all other groups (except rBCG::AB) versus rBCG::AB with a *P* < 0.05 in lung bacterial load; PBS versus rBCG::AB, rBCG::261 versus rBCG::AB, and all other groups (except rBCG::261) versus rBCG::261 with a *P* < 0.05 in spleen bacterial load. At 18 w after challenge, PBS versus rBCG::261, rBCG::85B versus rBCG::261, rBCG::AB versus rBCG::261, and all other groups (except rBCG::AB) versus rBCG::AB with a *P* < 0.05 in lung bacterial load; only PBS versus rBCG::261 and PBS versus rBCG::AB with a *P* < 0.05 in spleen bacterial load.

Six weeks after immunization, C57BL/6 mice were challenged with virulent *M. tuberculosis* strain, in order to determine the protective efficacy of different vaccines. Four weeks later, mice were killed and bacterial load in the lung and spleen of each mouse was analyzed. A challenge led to the highest bacterial load in the lung and spleen in PBS control animals (Figure 6). Vaccination with different rBCG strains strongly reduced bacterial load in the lung and spleen in C57BL/6 mice. rBCG::AB resulted in the most significant decrease of the bacterial load in the lung (*P* < 0.05), when compared with other three rBCG strains vaccinated mice at both 4 and 18 weeks after infection. Moreover, bacterial load of *M. tuberculosis* in lung tissue of rBCG::AB group at 18 week also decreased when compared with that of 4 weeks after infection. rBCG::AB also inhibited the growth of *M. tuberculosis* more significantly in the spleen than rBCG::261 vaccinated C57BL/6 mice at 4 weeks after infection. However, there was no statistical difference in bacterial load in spleen of all rBCG vaccinated C57BL/6 mice at 18 week. 

### 3.6. Evaluation of Pathological Changes in Lung Tissue

**Figure 7:**
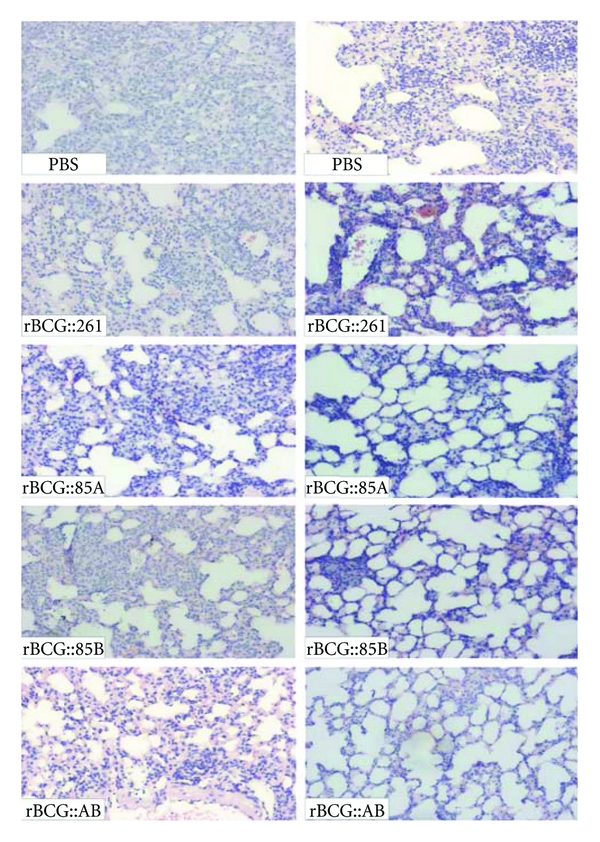
Representative lung pathology of C57BL/6 mice after challenge. Vaccinated C57BL/6 mice were challenged i.v. with 10^6^ CFU virulent *M. tuberculosis* H37Rv strain. Four and 18 weeks after infection, lung tissue sections from different vaccine groups were prepared for HE staining (×40).

Lung tissue from different groups of C57BL/6 mice was fixed, sectioned, and stained with HE stain for assessment of pathological changes (Figure 7). Four weeks after the challenge infection, there were the most extensive serious pathological changes in the lung from mice of PBS control group, which showed severe interstitial pneumonia and intense inflammation throughout the lung. Eighteen weeks later, the largest number of granuloma-like changes was found in the lung section from PBS control mice. More serious pathological changes with interstitial pneumonia and intense inflammation were also observed in the lung from rBCG::261, rBCG::85A, and rBCG::85B vaccinated groups at 4 weeks after infection than at 18 week after challenge. Comparatively, pathological changes were much less in the lung from mice vaccinated with rBCG::AB, which appeared to be intact with very limited lung inflammation at 4 or 18 week after infection. The result of acid-fast staining of lung tissue also supported the clear hierarchy of bacterial load in lung of different treated mice.

## 4. Discussion

Although BCG vaccine has been used to prevent against human TB since 1921, its efficacy is highly variable in protection against adult pulmonary TB [27]. Development of a new vaccine with a superior protection over BCG has become essential and urgent. Up to now, eleven vaccine regimens, including two recombinant BCG strains (rBCG30 over-expressing Ag85B and rBCG_ UreC:Hly), have progressed in different stages of human clinical trials [28]. rBCG candidate vaccines should induce more long-lasting protection than the parent BCG against TB, thus providing stronger protection to the adult after neonatal vaccination. In this study, we constructed the recombinant BCG strain (rBCG::AB) overexpressing the important immunodominant antigens both Ag85A and Ag85B of *M. tuberculosis* and evaluated the immunogenicity and protective efficacy of rBCG::AB in mice. Our study demonstrated that rBCG::AB strain could provide the most significant enhanced and enduring protection against virulent *M. tuberculosis* infection than the parental BCG vaccine, or rBCG strains overexpressing single antigen Ag85B or Ag85A alone, as evidenced by the lowest bacterial load in lung tissue and the lest change in lung pathology after short-term or long-term challenge infection with virulent *M. tuberculosis*. Thus, it is clear that BCG overexpressing of multiple antigens could provide more enhanced protective efficacy than BCG only overexpressing single antigen.

Many factors could influence the efficacy of recombinant BCG strains [29]. On the one hand, identification of promising antigen candidates for rBCG construction plays a critical role. Not all antigens, confirmed by DNA vaccination or other vaccine forms, functioned the same by recombinant BCG technology, such as 19 kDa [30, 31] and 27 kDa lipoprotein [32]. Previously, our and others studies demonstrated that DNA vaccines encoding Ag85A or Ag85B could provide a certain degree of protection against TB in mouse model [26, 33] and both antigens can be considered as the most promising candidate antigens for future TB vaccine development. Consistent with the previous report of rBCG30 [2–5], rBCG::85B overexpressing antigen Ag85B could inhibit the growth of *M. tuberculosis* in the lung more significantly than rBCG::261 18 weeks after challenge. The growth of *M. tuberculosis* in the lung was inhibited significantly in mice vaccinated with rBCG::85A overexpressing antigen Ag85A. Moreover, rBCG::85A also conferred the more significant protection in the spleen than rBCG::261 4 weeks after challenge. Therefore, our results have demonstrated that both antigens Ag85A and Ag85B might be suitable for vaccine candidates for enhancing immunogenicity and protective efficacy of BCG against virulent *M. tuberculosis* challenge in humans.

On the other hand, the methods for antigen expression in BCG also could influence the efficacy of rBCG candidate vaccines. Recombinant BCG secreting ESAT-6 strain, under Hsp60 promoter and Ag85B signal peptide, was found to provide no enhanced protection compared with the parental BCG [34]. However, when arranged and secreted in its native form, ESAT-6 secreting recombinant BCG strain could provide more significant protection [35]. Both antigens Ag85A and Ag85B are the major secreted proteins in the culture filtrates of *M. tuberculosis* or BCG [36]. In this study, when the full-length sequences of *fbpA* or *fbpB* (including the flanking regulatory, promoter and signal peptide and mature peptide sequences) were cloned into the BCG, we confirmed that overexpression of Ag85A and Ag85B was secreted on the cell wall and in the culture supernatants of rBCG::AB strain. Antigen expressed secreted or linked to the mycobacterial cell membrane by BCG cells has been confirmed which could influence both the timing of the immune response and the pathway by which antigen is presented to the immune system [29]. The presentation pathways, for example, via the MHC class II presentation pathway, determined the nature of the subsequent cellular and humoral immune responses induced by rBCG. CD4+ T cells and IFN-*γ* responses are important components of protection against *M. tuberculosis *[37–39]. After rBCG::AB immunization, Ag85A and Ag85B antigen-specific IFN-*γ* secreting splenocytes were significantly increased and more cells were produced than those of rBCG::261 or rBCG strains overexpressing single protein during the entire period, which may contribute to the enhanced and enduring protection. Screening and evaluating more immunodominant antigens by recombinant BCG technology will facilitate the rational design of rBCG vaccine for TB prevention.

## 5. Conclusion

In this study, our results demonstrated the vaccination of C57BL/6 mice with rBCG::AB overexpressing proteins Ag85A and Ag85B could result in the most significant protection against challenge with virulent *M. tuberculosis* H37Rv when compared with rBCG::261, rBCG::85A, or rBCG::85B alone. Thus, our study indicates that rBCG::AB may be a promising vaccine candidate against TB and should be used for the further preclinical test.
